# Generation of Vertebra Micro-CT-like Image from MDCT: A Deep-Learning-Based Image Enhancement Approach

**DOI:** 10.3390/tomography7040064

**Published:** 2021-11-12

**Authors:** Dan Jin, Han Zheng, Qingqing Zhao, Chunjie Wang, Mengze Zhang, Huishu Yuan

**Affiliations:** 1 Department of Radiology, Peking University Third Hospital, Beijing 100191, China; jindan1418@bjmu.edu.cn (D.J.); qqzhao@pku.edu.cn (Q.Z.); chunjiewang17@126.com (C.W.); zmzforever@pku.edu.cn (M.Z.); 2 School of Traffic and Transportation, Beijing Jiaotong University, Beijing 100044, China; hanzheng@bjtu.edu.cn

**Keywords:** computed tomography, osteoporosis, vertebra, trabecular bone, deep learning, structure analysis

## Abstract

This paper proposes a deep-learning-based image enhancement approach that can generate high-resolution micro-CT-like images from multidetector computed tomography (MDCT). A total of 12,500 MDCT and micro-CT image pairs were obtained from 25 vertebral specimens. Then, a pix2pixHD model was trained and evaluated using the structural similarity index measure (SSIM) and Fréchet inception distance (FID). We performed subjective assessments of the micro-CT-like images based on five aspects. Micro-CT and micro-CT-like image-derived trabecular bone microstructures were compared, and the underlying correlations were analyzed. The results showed that the pix2pixHD method (SSIM, 0.804 ± 0.037 and FID, 43.598 ± 9.108) outperformed the two control methods (pix2pix and CRN) in enhancing MDCT images (*p* < 0.05). According to the subjective assessment, the pix2pixHD-derived micro-CT-like images showed no significant difference from the micro-CT images in terms of contrast and shadow (*p* > 0.05) but demonstrated slightly lower noise, sharpness and trabecular bone texture (*p* < 0.05). Compared with the trabecular microstructure parameters of micro-CT images, those of pix2pixHD-derived micro-CT-like images showed no significant differences in bone volume fraction (BV/TV) (*p* > 0.05) and significant correlations in trabecular thickness (Tb.Th) and trabecular spacing (Tb.Sp) (Tb.Th, R = 0.90, *p* < 0.05; Tb.Sp, R = 0.88, *p* < 0.05). The proposed method can enhance the resolution of MDCT and obtain micro-CT-like images, which may provide new diagnostic criteria and a predictive basis for osteoporosis and related fractures.

## 1. Introduction

The spine, which consists of vertebrae, is the main load-bearing component of the body, and its skeletal status influences a person’s quality of life. Osteoporotic fractures, particularly vertebral fractures, can be associated with chronic disabling pain and even directly affect a person’s survival and life expectancy. Clinical diagnosis of osteoporosis and assessment of fracture risk are mainly based on the areal bone mineral density (BMD) of trabecular bone in the spine and/or hip observed using dual energy X-ray absorptiometry (DEXA) [1]. However, a number of clinical studies have demonstrated the limitations of BMD measurements. It has been recognized that BMD can account for only 60% of the variation in bone strength [2]. Recently, researchers found that concomitant deterioration of the bone structure, especially structural changes in trabecular bone, occurs with the loss of bone mass [3]. This deterioration and loss of bone mass both reduces bone quality and increases fracture susceptibility, indicating that bone structure also plays a key role in bone strength.

Microcomputed tomography (micro-CT), the gold standard for measuring bone microstructure, is an imaging system with exceptionally enhanced resolution (at the micron level) and can generate three-dimensional (3D) images of internal microstructures [4]. However, micro-CT scanners cannot be applied to materials larger than 10 cm in diameter (e.g., human torso), precluding their incorporation into in vivo imaging and diagnosis. Clinical multidetector computed tomography (MDCT) is widely used in the imaging diagnosis of spinal diseases, but it does not allow for accurate measurements of bone microstructure to be determined. Previously published in vitro studies have investigated the feasibility of using MDCT to measure bone structure, with some parameters exhibiting only a moderate correlation with that of micro-CT [5,6,7]. The trabecular bone thickness (Tb.Th) is approximately 100 microns, which is far less than the maximum resolution of MDCT images of approximately 200–500 microns [8]. Thus, the ideal imaging instrument for analyzing the structure of trabeculae needs to meet the requirement of a resolution lower than that of the thinnest trabeculae [9]. However, there is still a lack of suitable in vivo methods for measuring the microstructures of vertebrae. Therefore, we hope to find a method to enhance the resolution of MDCT images to obtain more image information about patients’ bone structure, which will help improve the accuracy of osteoporosis diagnosis and related fracture prediction.

In the medical imaging field, image enhancement methods have recently been used to improve the visualization of important details [10,11], for example, defects of retinal blood vessels [12] and indications of tuberculosis [13]. Essentially, there are three kinds of methods used in medical image enhancement: example-based methods [14], convolutional neural network (CNN)-based methods [15,16,17,18] and conditional generative adversarial network (CGAN)-based methods [19,20,21,22,23]. However, most of these methods can accommodate only mappings of local regions or low-resolution images and lack stability when high-resolution images are being evaluated [21]. To enhance vertebral images, with the goal of making accurate measurements of the bone microstructures, we needed to map the structure, orientation and other specific features of the trabecular bone between two sets of images (i.e., micro-CT and MDCT) with large resolution differences. Pix2pixHD [24], a CGAN-based method, consists of a coarse-to-fine generator and multiscale discriminators and is designed for the generation of high-detail and high-resolution images of more than 2048 × 1024, which fits our research needs. Therefore, we used pix2pixHD in our endeavor to enhance MDCT images of vertebrae.

In this study, intact vertebrae from human cadavers were imaged using clinical MDCT and micro-CT imaging protocols to (1) take micro-CT images as the gold standard and to regard corresponding MDCT images as inputs to train the pix2pixHD model to enhance vertebral images and obtain micro-CT-like images; (2) use objective image quality metrics to compare the performance of the proposed model with that of two other models named pix2pix and CRN to determine which method is the most suitable for enhancing vertebral images; (3) compare the difference between pix2pixHD-derived micro-CT-like images and micro-CT images by a subjective assessment method to evaluate the quality of the micro-CT-like images and (4) assess the accuracy of trabecular bone structure metrics generated from pix2pixHD-derived micro-CT-like images using micro-CT images as the gold standard to further validate that the proposed method is clinically applicable.

## 2. Materials and Methods

### 2.1. Specimens

This study was performed with 5 sets of lumbar spines (between segments L1 and L5, including 25 vertebrae) harvested from 5 formalin-fixed human cadavers (3 males and 2 females; mean age, 75 years; age range, 68–88 years). The donors had dedicated their bodies for educational and research purposes to the local Institute of Anatomy prior to death, in compliance with local institutional and legislative requirements. Lumbar vertebrae with significant compression fractures, bone neoplasms or other causes of significant bone destruction were excluded. All 25 specimens were included in the experiment. The lumbar spine with surrounding muscle was cut into individual segments using a band saw, with pedicle and appendix structures preserved as much as possible. The samples were immersed in phosphate-buffered saline (PBS) solution at 4 °C for 24 h prior to scanning to minimize trapped gas. The study protocol was reviewed and approved by the local institutional review boards.

### 2.2. Imaging Techniques

The specimens were scanned by micro-CT (Inveon, Siemens, Erlangen, Germany) and MDCT (SOMATOM Definition Flash, Siemens, Erlangen, Germany). The parameters of micro-CT imaging were 80 kVp/500 mAs, the field of view on the xy plane was 80 × 80 mm^2^, the standard matrix size used was 1536 × 1536 pixels, the number of slices was 1024 at an effective pixel size of 52 μm and the exposure time was 1500 ms in each of the 360 rotational steps. The MDCT imaging parameters were 120 kVp/250 mAs, the field of view was 100 × 100 mm^2^, the slice thickness was 0.6 mm, the slice interval was 0.1 mm, pitch was 0.8 and the standard matrix size used was 512 × 512 pixels. The two scans provided stack images on the axial plane that covered the entire vertebrae.

### 2.3. Image Co-Registration

The independent acquisition of the two scanning methods causes MDCT and micro-CT images to be mismatched. To obtain micro-CT-like images, the first step is to achieve slice matching between MDCT and micro-CT axial images [25,26]. The scanned micro-CT slice interval was approximately 0.05 mm, and the MDCT slice interval was approximately 0.1 mm. For a sample of any of the 25 vertebrae, after removing images with incomplete vertebral structures and images involving the upper and lower endplates, we selected an area of 2.5 cm in height on the vertebra, obtaining approximately 500 micro-CT images and 250 MDCT images. There were twice as many micro-CT images as MDCT images.

**Figure 1:**
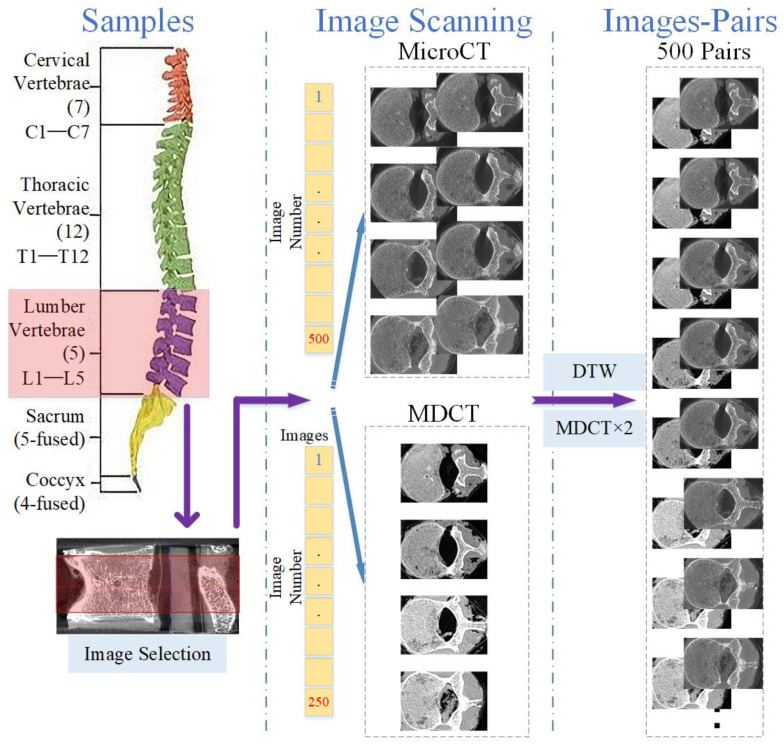
Illustration of image co-registration for micro-CT and MDCT images.

Subsequently, the MDCT and micro-CT images were compared one by one, and the best image mappings were obtained by the dynamic time warping (DTW) algorithm [27] and scale-invariant feature transform (SIFT) [28]. Then, the MDCT images were doubled according to the mapping relationships to obtain MDCT and micro-CT image pairs. Applying the above method to each of the 25 vertebrae, a total of 25 × 500 = 12,500 image pairs could be obtained. The image pairs were stored in database_0. Figure 1 illustrates the process mentioned above.

### 2.4. Construction of Training Set and Testing Set

**Figure 2:**
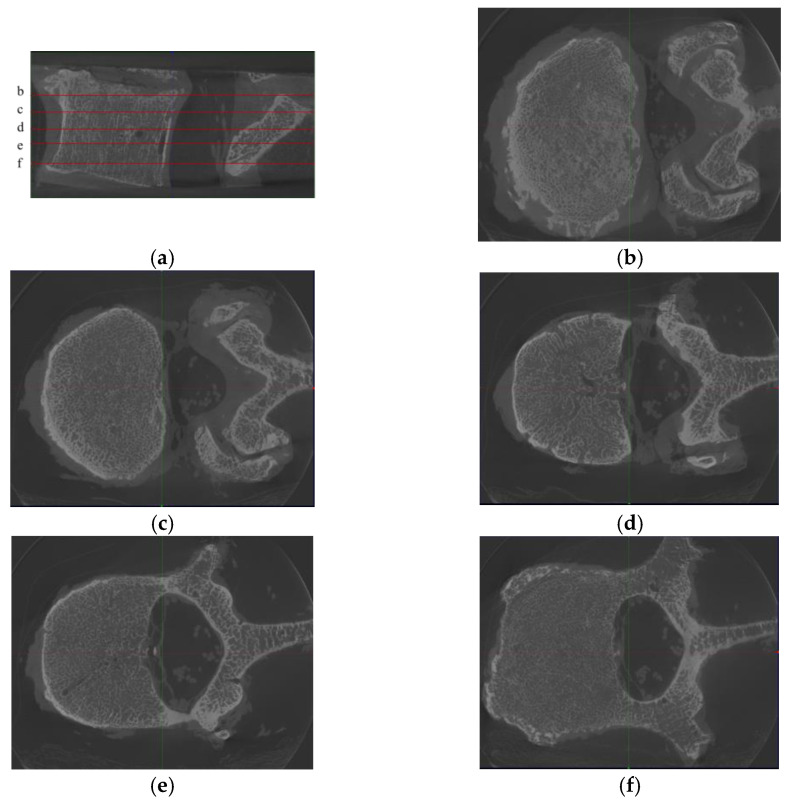
Samples from one L2 vertebra, where (**a**) is the sagittal position image of the L2 vertebra, with 5 noted slices (named **b**–**f**), and (**b**)–(**f**) are the corresponding axis position images. We found that although the images were from the same vertebra, the differences between the images of different slices were substantially large. Moreover, since the technique used in this paper is an image-to-image technique, there is no longer a holistic concept of “vertebra” in the training process but only discrete images.

In our study, we assumed that images from the obtained 12,500 image pairs can be treated as individual samples from the micro-CT domain and MDCT domain. In other words, the relationship between these samples and the vertebrae to which they belong was ignored during training and testing. This assumption is supported by the following reasons:

(1) Characteristics of the selected model. In this paper, we intended to map MDCT images to micro-CT-like images using an image-to-image method named pix2pixHD. This method is a supervised paired image learning method that maps images from the source MDCT domain to the target micro-CT domain and does not consider the continuity within the image domain. Image pairs are randomly selected for tuning the model during training, and no images of a particular vertebra are fed into the training as a set. In other words, in the framework of the selected technique, all image pairs are considered independent during training, and the correlation between different slices of images within a vertebra is ignored.
(2) Diversity within each vertebra. Due to the diversity of images at each slice inside vertebrae (see Figure 2), the images within a vertebra do not obey the same distribution. This diversity is even more pronounced in the presence of vertebral attachments. To better realize the training, we needed to use all pairs of images at all slices in vertebrae as the basic unit for model training.

Therefore, there was no “vertebra” in the training and testing processing but only image pairs. The sequential information can be further broken if the training set and test set are constructed by random sampling. The training and test sets obtained on this basis can be considered to be independent.

Based on the above analysis, we could obtain the test set and training set by random sampling. To prevent a certain slice of images from being trained, for any vertebra, 100 image pairs (20%) were randomly selected as the testing set, and the remaining 400 image pairs were used as the training set (80%) [29]. Random sampling ensured that continuous information was removed, and the training and test sets covered most parts of the vertebrae so that the trained model did not suffer from underfitting or overfitting. In this way, the 12,500 image pairs in database_0 were divided into training (dataset_training) and test (dataset_testing) sets.

### 2.5. Model Training

**Figure 3:**
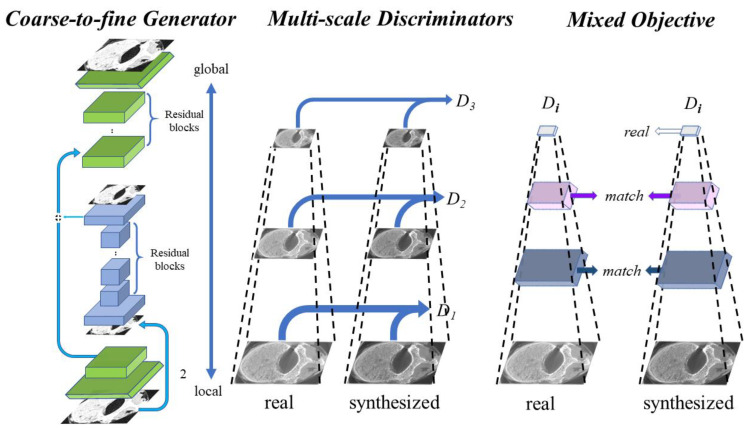
Architecture of the pix2pixHD model used, where the global generator consists of 3 components: a convolution front-end, a set of residual blocks, and a transposed convolutional back-end. The local generator also consists of 3 components: a convolutional front-end, a set of residual blocks, and a transposed convolutional back-end. The multiscale discriminators consist of three identically structured networks.

Pix2pixHD [24] is a model based on a CGAN that can generate high-resolution micro-CT-like images given the input MDCT images by finding the complex mapping function. The framework of pix2pixHD consists of a coarse-to-fine generator and multiscale discriminators. The coarse-to-fine generator contains a global generator network and a local enhancer network, where the global generator network focuses on coarse and global features of images (such as external contours and geometric structures) and the local enhancer network focuses on local details (such as the texture and direction of bone trabeculae). Similar ideas but different architectures can be found in [30,31,32]. These multiscale discriminators are designed for training the coarse-to-fine generator using three identically structured networks focusing on different scales of details. The network framework of the pix2pixHD model is shown in Figure 3.

The pix2pixHD model was trained in the PyTorch platform on a Windows Server 2019 workstation with two Nvidia A6000 graphics processing units (GPUs). The batch size was set as 10. The maximum number of epochs was set as 200, and there were 200 iterations in each epoch. We compared our method with two other mature methods: CRN [33] and pix2pix [21]. We trained these two models with their default settings.

### 2.6. Objective Assessment of Image Quality

After training, the pix2pixHD model was validated by objective metrics based on the designed testing set (dataset_testing), as were the pix2pix and CRN methods. The objective metrics are described below.

Structural similarity index measure (SSIM) [34]: The SSIM computes the perceptual distance between micro-CT-like images and the gold standard (i.e., micro-CT images). In this paper, we used the simplified version of the SSIM:

$$\begin{array}{r} SSIM\left(x,y\right)=\frac{\left(2\mu_{x}\mu_{y}+C_{1}\right)\left(2\sigma_{xy}+C_{2}\right)}{\left(\mu_{x}^{2}+\mu_{y}^{2}+C_{1}\right)\left(\sigma_{x}^{2}+\sigma_{y}^{2}+C_{2}\right)} \end{array}$$

where $\mu_{x}$ and $\mu_{y}$ are the average values of input images *x* and *y*, respectively. $C_{1}={\left(K_{1}L\right)}^{2}$ and $C_{2}={\left(K_{2}L\right)}^{2}$, where $K_{1}\;\mathrm{and}\;K_{2}\ll 1$ are small constants (the default values of $k_{1}$ and $k_{2}$ are 0.01 and 0.03, respectively), and L is the dynamic range of the pixel values (255 for 8-bit grayscale CT images).

Fréchet inception distance (FID) [35]: The FID measures the distance between a generated micro-CT-like image and the corresponding micro-CT image by extracting a feature vector with 2048 elements by a trained Inception-V3 model. The FID formula is as follows:

$$\begin{array}{r} FID={∥\mu_{r}-\mu_{g}∥}^{2}+Tr\left(C_{r}+C_{g}-2{\left(C_{r}C_{g}\right)}^{1/2}\right) \end{array}$$

where $\mu_{r}$ and $\mu_{g}$ are the mean values of the features of the real and generated images, respectively, and $C_{r}$ and $C_{g}$ are the covariance matrices of the real and generated images, respectively.

These two indexes evaluate the similarity between two images from different perspectives. The SSIM tends to evaluate similarity in terms of structure, and higher SSIM indicates higher similarity of the images [36]. In contrast, the FID tends to evaluate similarity in terms of details, and a lower FID indicates a higher similarity of the images [35]. The above two objective metrics validated the generated micro-CT-like images from a computer imaging perspective. By comparing the two metrics from the results of the three methods (pix2pixHD, pix2pix and CRN), we could ascertain the effectiveness of the three methods and determine which method better enhances vertebral images.

### 2.7. Subjective Assessment of Image Quality

Subjective assessment of image quality was performed by three radiologists (Observer 1, J.D., 6 years of experience in musculoskeletal imaging; Observer 2, Z.Q., 5 years of experience in musculoskeletal imaging; Observer 3, W.C., 3 years of experience in musculoskeletal imaging) through image scoring. The detailed experimental operation was as follows: to prevent visual fatigue of the observers which could impact the fairness of the scoring results, we randomly selected 30 micro-CT images and 30 pix2pixHD-derived micro-CT-like images and sorted them into a sequence as an experimental collection. Each image was assigned a unique identification number. These sequences were anonymized and presented to the three observers independently in a blinded and random fashion. To provide comparable results, all images were displayed using the same graphics software, and all images were consistent in size, window level and width. Contrast was rated on a 3-point scale, and noise, sharpness, shadow and texture were rated on a 5-point scale to assess image quality. These ratings are further described in Table 1.

### 2.8. Assessment of the Trabecular Bone Microstructure

**Figure 4:**
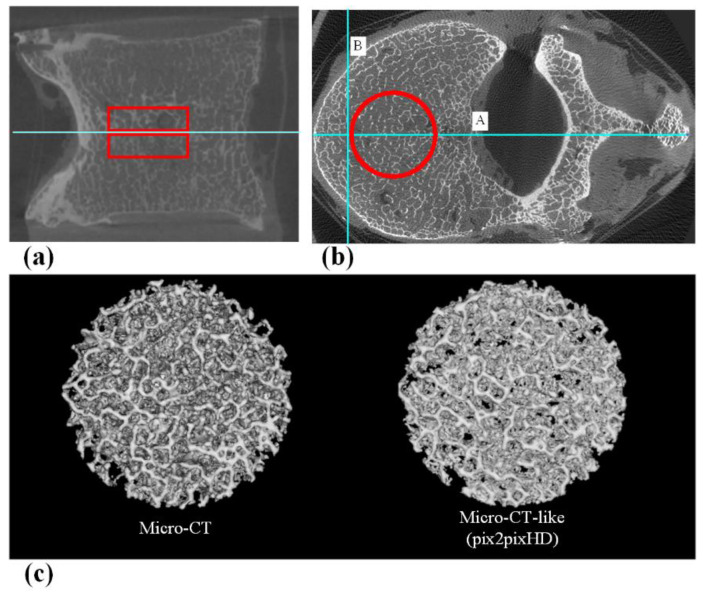
(**a**) The sagittal position of the VOI, which includes two areas 5 mm above and below the central slice. (**b**) The axial position of the VOI. First, the vertebral body central axis line A (Horizontal positioning line) was drawn, and then, line B (Vertical positioning line) was drawn perpendicular to line A at 5 mm inside the intersection of line A and the anterior edge of the vertebral body. Using the intersection of lines A and B as the tangent point, a cylindrical VOI with a diameter of 15 mm was outlined. (**c**) 3D reconstructed VOI of micro-CT and micro-CT-like images.

To measure the bone microstructure, we needed to obtain continuous axial images to form a cylindrical volume of interest (VOI). After training the model, we inputted all the original MDCT images of the 25 vertebrae from database_0 into the pix2pixHD model to obtain continuous micro-CT-like images. Then, we selected micro-CT-like images with the original micro-CT images of the 25 vertebrae. Then, two cylindrical VOIs (approximately 15 mm in diameter and 5 mm in height) for each vertebra (*n* = 50 in total) were defined on both the micro-CT and micro-CT-like images. The positioning of the VOI can be found in Figure 4. The same VOI setting was also used for MDCT images to calculate bone structure parameters as a control group.

Trabecular microstructure analysis of the micro-CT-like and micro-CT images was performed using the BoneJ plug-in [37] in Fiji [38]. Fiji is a distribution of the image processing package ImageJ2 (National Institutes of Health, USA) [39,40]. The micro-CT-like images of the vertebrae were processed in conjunction with the micro-CT images as 8-bit stack maps in Fiji software. The micro-CT and micro-CT-like grayscale image pairs were binarized into bone and marrow phases using a global (histogram-derived) thresholding method named the IsoData algorithm [41]. The underlying assumption of this method is that the histogram intensity distribution is bimodal, exhibiting bone and background peaks. The midpoint between the two peaks was used as the threshold value. Then, the following structural parameters were calculated: bone volume fraction (BV/TV), trabecular thickness (Tb.Th) and trabecular spacing (Tb.Sp). BV/TV was derived through simple voxel counting. In this method, all the foreground voxels were counted, and all voxels were assumed to represent bone; then, the number of foreground voxels was compared to the total number of voxels in the image. Tb.Th and Tb.Sp were calculated without model assumptions as direct measures. Foreground voxels were considered to be trabeculae, and background voxels were regarded as the spacing [42]. BoneJ was used to calculate the mean and the standard deviation of the Tb.Th or Tb.Sp directly from pixel values in the resulting thickness map.

### 2.9. Statistics

The Kolmogorov–Smirnov test was used to analyze normality, and the Levene test was used to analyze the homogeneity of variance among the measurement data. Data showing a Gaussian distribution are reported as the mean ± standard deviation. For objective image analysis, because the data did not satisfy homogeneity of variance, the Kruskal–Wallis test was used to assess the difference in the SSIM and FID for the three methods. For the subjective assessment, Kendall’s coefficient of concordance (Kendall’s W) was calculated to evaluate interobserver agreement for each subjective image evaluation score of 5 aspects. We considered Kendall’s W values of less than 0.20 to be indicative of poor agreement, values between 0.20 and 0.40 to indicate fair agreement, values between 0.60 and 0.80 to indicate moderate agreement and values greater than 0.80 to indicate excellent agreement. Then, the Mann–Whitney U test was performed to compare the subjective assessment scores between micro-CT and pix2pixHD-derived micro-CT-like images. For trabecular bone microstructure analysis, the paired Student’s t-test was used to determine the statistical significance of differences between micro-CT and micro-CT-like images for each structural parameter. Parameters derived from the micro-CT and micro-CT-like images were correlated using Pearson’s correlation coefficient. These statistical analyses were performed using SPSS 26.0 software (SPSS Inc., Chicago, IL, USA), and a *p*-value < 0.05 was considered statistically significant.

## 3. Results

**Figure 5:**
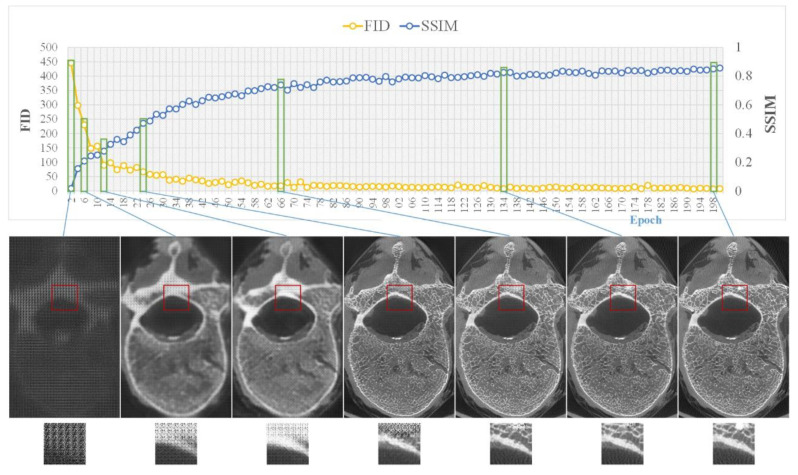
Model training, where the top side shows the changes in the two metrics (i.e., SSIM and FID) during training and the bottom side shows sample images from several key epochs.

The training process of pix2pixHD required 653 min in total, which is close to the time required for the training process of pix2pix (603 min) and CRN (698 min). Figure 5 shows the evolution of the SSIM and FID of pix2pixHD during training.

### 3.1. Objective Assessment of Micro-CT-like Image Quality of the Three Evaluated Methods

**Figure 6:**
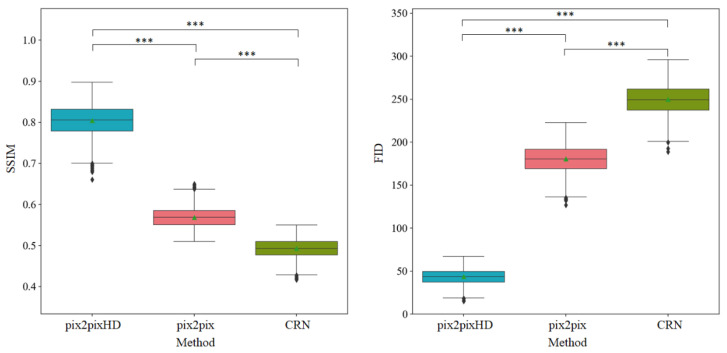
Objective assessment metrics comparison of three methods. Horizontal lines show the significant results of Kruskal–Wallis tests. *** statistical significance with *p* < 0.001.

Figure 6 shows the SSIM and FID metrics between the sets of micro-CT images and micro-CT-like images generated from the three methods. The mean SSIM values of pix2pixHD-, pix2pix- and CRN-derived micro-CT-like images were 0.804 ± 0.037, 0.568 ± 0.025 and 0.490 ± 0.023, respectively, and the differences were statistically significant (*p* < 0.001 for both). Additionally, the mean FID of pix2pixHD-derived micro-CT-like images was 43.598 ± 9.108, which was significantly smaller than that of the pix2pix (180.317 ± 16.532) and CRN (249.593 ± 17.993) methods (*p*  <  0.001 for both).

### 3.2. Subjective Assessment of pix2pixHD-Derived Micro-CT-like Image Quality

The summary of subjective assessment scores and Kendall’s W in Table 2 shows the interobserver agreements on five aspects in pix2pixHD micro-CT-like images and micro-CT images. The subjective scoring of shadow was perfectly consistent. In addition, the Kendall’s W values of the other four aspects were between 0.800 and 0.959 (*p* < 0.001), demonstrating excellent interobserver agreement. Then, we averaged the scores to analyze the differences between two sets of images, as shown in Table 3. The noise, sharpness and trabecular bone texture scores of pix2pixHD-derived micro-CT-like images were slightly lower than those of micro-CT images (*p* = 0.002, *p* = 0.004 and *p* = 0.013, respectively). In addition, there was no significant difference between the subjective scores of the two sets of images in terms of contrast and overlapping shadow (*p* = 0.716 and *p* = 1.000, respectively). In particular, in terms of overlapping shadows, the mean subjective scores for both methods were five points, indicating that no significant overlap shadow existed in either set of images.

### 3.3. Assessment of Trabecular Bone Microstructure with pix2pixHD-Derived Micro-CT-like Images and Micro-CT Images

As shown in Table 4, comparison of the trabecular bone microstructure parameters obtained from pix2pixHD-derived micro-CT-like images with those from micro-CT images showed that there were no significant differences in BV/TV (*p* = 0.101). The Tb.Th and Tb.Sp of micro-CT-like images (0.179 ± 0.027 and 0.758 ± 0.479 mm, respectively) were significantly lower than those of the corresponding micro-CT images (0.220 ± 0.012 and 0.934 ± 0.126 mm, respectively) (*p* < 0.01).

The correlation coefficients (R) between the micro-CT-like image- and micro-CT-derived trabecular bone structure parameters are also shown in Table 4. The values of BV/TV, Tb.Th and Tb.Sp determined from the micro-CT-like images showed high correlations with those determined from the micro-CT images, and all correlations were significant (*p <* 0.001).

We also compared the bone microstructure parameters obtained from MDCT images with those from micro-CT images. The results are shown in Table 5. We found that the BV/TV and Tb.Th values of MDCT images (0.320 ± 0.067, 0.680 ± 0.079 mm) were significantly higher than those of the corresponding micro-CT images (*p* < 0.001). However, the Tb.Sp (0.870 ± 0.140 mm) of MDCT images was lower than that of micro-CT images (*p* < 0.001).

The correlation coefficients (R) between the MDCT and micro-CT-derived trabecular bone structure parameters are also shown in Table 5. The values of BV/TV, Tb.Th and Tb.Sp determined from the MDCT images showed moderate correlations with those determined from the micro-CT images (*p <* 0.001).

## 4. Discussion

In this paper, we used a deep-learning-based method, pix2pixHD, to find mappings between MDCT and micro-CT axial images to generate micro-CT-like images of vertebrae. To our knowledge, integrating image mapping and texture accuracy enhancement between two sets of images with very different textures and details, such as MDCT images and micro-CT images, is still a challenge; additionally, this is the first attempt to map micro-CT and MDCT images using the deep-learning-based pix2pixHD method.

By comparing the performance of the three methods regarding the generated images using objective image assessment metrics, it was demonstrated that the pix2pixHD method resulted in superior micro-CT-like images compared to the other two methods, with sufficient similarity between the generated images and the corresponding micro-CT images. This similarity was reflected not only in the overall vertebral body but also in the local details and anatomical subtleties of the images. The reason the pix2pixHD method outperformed the other methods is that it adopts a multiscale generator and discriminators, considering the overall structure and local details. In contrast, the CRN and pix2pix models were not designed for the high-resolution and high-detail medical image enhancement problem; they do not have an adequate field of view and have severe overlapping shadow and blurring problems when processing high-resolution images [24].

All three observers had high agreement on all subjective image quality scores and concluded that the contrast and overlapping shadow scores of pix2pixHD-derived micro-CT-like images were not significantly different from those of micro-CT images. This means that the generated images were excellent in both aspects. This result arises because pix2pixHD’s generator and discriminator were both built using a multiscale architecture and can generate high-detail and high-resolution images with a resolution of more than 2048 × 1024, which covered our image scope completely.

**Figure 7:**
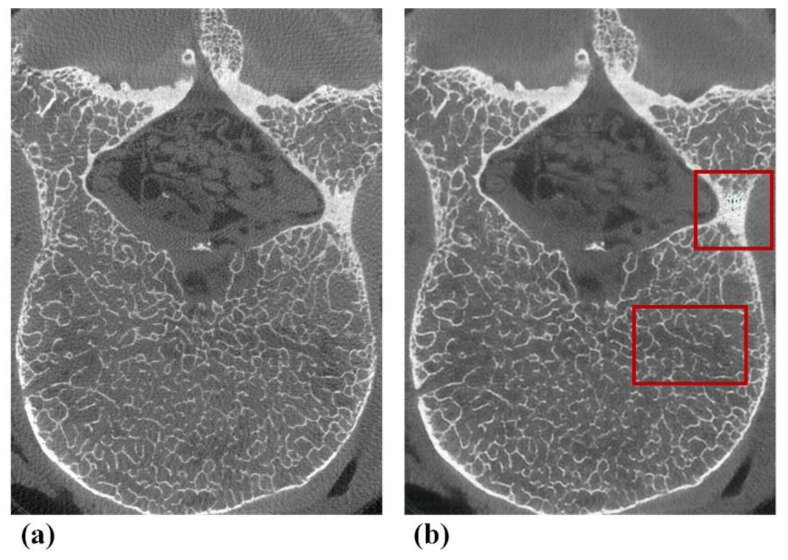
Typical examples of defects of pix2pixHD-derived micro-CT-like images found in subjective assessments. (**a**) Gold-standard micro-CT image. (**b**) Corresponding pix2pixHD-derived micro-CT-like image with noise in the appendage area and a slightly blurred area in the vertebral body.

Micro-CT-like images also have some shortcomings, with a slightly deficient performance in terms of noise, sharpness and ability when visualizing trabecular bone texture compared to micro-CT images. We reviewed our micro-CT-like images with relatively low noise scores and found that noise was mainly found in the vertebral appendages (including the pedicles and laminae), as shown in Figure 7, which are characterized by a thicker bone cortex or markedly heterogeneous increases in bone density at localized positions. This outcome may be due to the complex interleaving of pixels representing bone contained in the abovementioned regions. The objective function [43] used by the model was insensitive to noise in this case. Fortunately, osteoporosis is mainly associated with the vertebral body, and noise at the above anatomical positions does not directly affect the accuracy of measurement of the bone structure of the vertebral body. Nevertheless, the pedicle is the clinical entry point for pedicle screws in spinal decompression and fixation fusion, especially in posterior internal fixation systems. Furthermore, studies have demonstrated that the bone quality of this component determines the stability of internal fixation [44,45,46]. Hence, in the future, we plan to use more auxiliary means to improve the accuracy of bone structure in vertebral appendages.

In addition, the observers subjectively determined the sharpness and trabecular texture scores of micro-CT-like images to be lower than those of micro-CT images (*p* < 0.001), which is consistent with the trend of our objective metric results (SSIM and FID) and trabecular bone measurement results (Tb.Th and Tb.Sp). This result arises because the method used is based on image-by-image mappings with insufficient consideration of the correlation between adjacent images. This caused the bone trabecular details to have unreasonable missing and abnormal textures, which reduced the corresponding score in the subjective evaluation. To solve the above problems, we need to increase the number of samples, build models that can extract association information between images and optimize the parameters of the training models in future work.

Regarding all trabecular bone structural measurements (BV/TV, Tb.Th and Tb.Sp) in our study, the correlation of their values computed from micro-CT and pix2pixHD-derived micro-CT-like images was very high (R > 0.88) and better than the correlation computed from micro-CT and MDCT images. The mean values of the measurements in our study were lower than those of the gold standard. Previously published in vitro studies on the feasibility of bone structure measurements using MDCT on vertebral bodies reported similar results for BV/TV. Issever et al. [5] reported a correlation coefficient of 0.86 (coefficient of determination, R^2^) for BV/TV measured in vertebrae specimens. However, the correlation between Tb.Th and Tb.Sp in the results of Issever et al. [5] was considerably weaker and rare (R^2^ = 0.19–0.26), which is consistent with our MDCT image results. Guha [6] and Chen [7] explored the correlation between trabecular bone structural measurements of MDCT and the corresponding micro-CT images using in vitro tibial and distal radius specimens. Although the anatomical positions of the study specimens were different, the correlation coefficients of Tb.Th and Tb.Sp was also relatively moderate (R < 0.80, Pearson). In addition, note that the mean values of MDCT-derived Tb.Th measured by the existing studies were greater than those of the gold standard, which is the same as our MDCT images but the opposite of the patterns we found from micro-CT-like images. Scholars have concluded that [5,7] this is a result of the relatively low image resolution of MDCT, causing the thinner trabeculae to be lost in the images and the trabecular network to be blurred. Unlike existing studies, our method can recover trabecular bone with widths smaller than the maximum resolution of MDCT by modeling the implicit mapping relationships in MDCT and micro-CT images. Through this method, we obtained Tb.Th and Tb.Sp values that were extremely close to those of the gold standard (R > 0.90). Notably, the bone structure metrics derived from our micro-CT-like images were lower than those of the gold standard. This is mainly because our trained pix2pixHD model still has some deficiencies in the extraction of image features during the generation of the map images, making the grayscale values of the pixels in and around the bone trabeculae fluctuate. This fluctuation directly affected the bone structure measurement process; in particular, it caused local disappearance, fragmentation and displacement of trabecular bone during the binarization process. The thickness of trabecular bone was reduced, and the number of bone trabeculae was increased.

In summary, our chosen method is more suitable for the task of generating high-resolution micro-CT-like images than previous methods are. Nevertheless, prior to implementation in clinical practice, the following improvements should be made in future studies. Firstly, the relationship between images needs to be captured by a 3D mapping model. Thus, the fineness of the bone trabecular texture can be further enhanced. Secondly, the relationship between bone structure metrics and bone biomechanical metrics needs to be analyzed. In the future, we plan to perform mechanical experiments on bone samples to determine the relationship between the bone structural metrics of generated micro-CT-like images and bone strength in a more detailed way. This relationship could be used to further enhance the significance of bone structural metrics studies for clinical applications, such as the diagnosis of osteoporotic fragility fractures.

Continued increases in life expectancy are predicted to increase the population with osteoporosis, and associated fracture rates are expected to increase as well. Therefore, it is essential to identify fracture risks to plan therapeutic interventions and monitor treatment responses. In addition, as the age of the population undergoing spinal instrumentation increases, clinicians need to consider bone quality more carefully than ever before and tailor surgical techniques to optimize patient outcomes and reduce the probability of postoperative complications [47]. Although our results are currently at the in vitro stage, with the expansion of the sample size, the inclusion of in vivo experiments and the maturation of the deep learning algorithm, it will be possible to obtain more accurate bone structural parameters while performing conventional CT scans in the future. Additionally, the bone density and bone structure measurements of vertebrae can be obtained simultaneously through the use of a commercial calibration phantom during MDCT scanning. These composite metrics may provide a new predictive basis for osteoporotic fractures and a new reference for surgical planning and drug selection.

## 5. Conclusions

In this paper, we applied a deep-learning-based image enhancement method that can generate full-sized high-resolution images from MDCT. This fully automatized deep-learning-based vertebral bone enhancement method performs better than other methods. Moreover, this method can make full use of MDCT images to accurately measure vertebral bone structure, which may provide new diagnostic criteria and a predictive basis for osteoporosis and related fractures and may provide a new reference for osteoporosis treatment and prevention.

## Figures and Tables

**Table 1:** Scoring method for the subjective assessment.

	Metrics	Scoring
1	Contrast between the trabecular bone and bone marrow	1. Too high or too low and unacceptable; 2. High or low but acceptable; 3. Optimal
2	Existence of noise	1. Severe and unacceptable; 2. Marked but acceptable; 3. Moderate; 4. Mild; 5. None or minimal
3	Sharpness of the trabecular bone	1. Severe blurring of the images and unacceptable; 2. Marked blurring of the images but acceptable; 3. Moderate blurring of the images; 4. Mild blurring of the images; 5. None or minimal blurring of the images
4	Obvious overlapping shadows	1. Severe and unacceptable; 2. Marked but acceptable; 3. Moderate; 4. Mild; 5. None or minimal
5	Natural shape of the trabecular bone texture	1. Poor and unacceptable; 2. Marked irregular and unnatural but acceptable; 3. Slightly irregular and unnatural; 4. almost defined and natural; 5. Completely defined and natural

**Table 2:** Interobserver agreement for subjective assessment scores of micro-CT and pix2pixHD-derived micro-CT-like images.

Indexes	Methods	Observer	Score					Kendall’s W	*p*-Value ^†^
			1	2	3	4	5		
Contrast	Micro-CT	Observer 1	0	7	23	\	\	0.912	<0.001
		Observer 2	0	6	24	\	\		
		Observer 3	0	5	25	\	\		
	Micro-CT-like	Observer 1	0	7	23	\	\	0.959	<0.001
		Observer 2	0	7	23	\	\		
		Observer 3	0	6	24	\	\		
Noise	Micro-CT	Observer 1	0	0	0	6	24	0.800	<0.001
		Observer 2	0	0	0	9	21		
		Observer 3	0	0	0	4	26		
	Micro-CT-like	Observer 1	0	0	4	8	18	0.938	<0.001
		Observer 2	0	0	5	5	20		
		Observer 3	0	0	5	8	17		
Sharpness	Micro-CT	Observer 1	0	0	0	4	26	0.817	<0.001
		Observer 2	0	0	0	9	21		
		Observer 3	0	0	0	8	22		
	Micro-CT-like	Observer 1	0	0	4	8	18	0.888	<0.001
		Observer 2	0	0	6	3	21		
		Observer 3	0	0	5	10	15		
Shadow	Micro-CT	Observer 1	0	0	0	0	30	0.000	1.000
		Observer 2	0	0	0	0	30		
		Observer 3	0	0	0	0	30		
	Micro-CT-like	Observer 1	0	0	0	0	30	0.000	1.000
		Observer 2	0	0	0	0	30		
		Observer 3	0	0	0	0	30		
Texture	Micro-CT	Observer 1	0	0	0	4	26	0.927	<0.001
		Observer 2	0	0	0	3	27		
		Observer 3	0	0	0	3	27		
	Micro-CT-like	Observer 1	0	0	3	6	21	0.908	<0.001
		Observer 2	0	0	2	4	23		
		Observer 3	0	0	2	4	24		

^†^ Calculated using Kruskal–Wallis test.

**Table 3:** Comparison of the subjective mean scores of micro-CT and pix2pixHD-derived micro-CT-like images.

	Micro-CT (*n* = 30)	Micro-CT-like (*n* = 30)	*p*-Value ^†^
Contrast	2.8 ± 0.402	2.78 ± 0.418	0.716
Noise	4.79 ± 0.410	4.46 ± 0.752	0.002
Sharpness	4.77 ± 0.425	4.43 ± 0.765	0.004
Shadow	5.00 ± 0.00	5.00 ± 0.00	1.000
Texture	4.89 ± 0.316	4.68 ± 0.615	0.013

^†^ Mann–Whitney U test for comparing subjective mean scores of micro-CT and micro-CT-like images.

**Table 4:** Trabecular bone structure parameters and correlation coefficient (R) of micro-CT and pix2pixHD-derived micro-CT-like images.

*n* = 50	Micro-CT	Micro-CT-like Images	*p*-Value ^†^	Correlation Coefficient of Micro-CT-like and Micro-CT Images (R)	*p*-Value ^‡^
BV/TV	0.180 ± 0.016	0.175 ± 0.034	0.101	0.920	<0.001
Tb.Th (mm)	0.220 ± 0.012	0.179 ± 0.027	<0.001	0.905	<0.001
Tb.Sp (mm)	0.934 ± 0.126	0.758 ± 0.479	0.002	0.885	<0.001

^†^ Paired Student’s *t*-test for comparing trabecular bone structure parameters of micro-CT and micro-CT-like images. ^‡^ Pearson’s correlation coefficient for verifying the correlation of trabecular bone structure parameters between micro-CT and micro-CT-like images.

**Table 5:** Trabecular bone structure parameters and correlation coefficients (R) of micro-CT and MDCT images.

*n* = 50	Micro-CT	MDCT	*p*-Value ^†^	Correlation Coefficient of MDCT and Micro-CT Images (R)	*p*-Value ^‡^
BV/TV	0.180 ± 0.016	0.320 ± 0.067	<0.001	0.514	<0.001
Tb.Th (mm)	0.220 ± 0.012	0.680 ± 0.079	<0.001	0.445	<0.001
Tb.Sp (mm)	0.934 ± 0.126	0.870 ± 0.140	<0.001	0.539	<0.001

^†^ Paired Student’s *t*-test for comparing trabecular bone structure parameters of micro-CT and MDCT images. ^‡^ Pearson’s correlation coefficient for verifying the correlation of trabecular bone structure parameters between micro-CT and MDCT images.

## Data Availability

Data sharing not applicable.
